# Crystal structure of 3-{[4-(2-meth­oxy­phen­yl)piperazin-1-yl]meth­yl}-5-(thio­phen-2-yl)-1,3,4-oxa­diazole-2(3*H*)-thione

**DOI:** 10.1107/S2056989016000992

**Published:** 2016-01-30

**Authors:** Monirah A. Al-Alshaikh, Hatem A. Abuelizz, Ali A. El-Emam, Mohammed S. M. Abdelbaky, Santiago Garcia-Granda

**Affiliations:** a Department of Chemistry, College of Sciences, King Saud University, Riyadh 11451, Saudi Arabia; b Department of Pharmaceutical Chemistry, College of Pharmacy, King Saud University, Riyadh 11451, Saudi Arabia; c Department of Physical and Analytical Chemistry, Faculty of Chemistry, Oviedo University-CINN, Oviedo 33006, Spain

**Keywords:** crystal structure, 1,3,4-oxa­diazole, thio­phene, piperazine, disorder, hydrogen bonding

## Abstract

The title compound, C_18_H_20_N_4_O_2_S_2_, is a new 1,3,4-oxa­diazole and a key pharmacophore of several biologically active agents. It is composed of a meth­yl(thio­phen-2-yl)-1,3,4-oxa­diazole-2(3*H*)-thione moiety linked to a 2-meth­oxy­phenyl unit *via* a piperazine ring that has a chair conformation. The thio­phene ring mean plane lies almost in the plane of the oxa­diazole ring, with a dihedral angle of 4.35 (9)°. The 2-meth­oxy­phenyl ring is almost normal to the oxa­diazole ring, with a dihedral angle of 84.17 (10)°. In the crystal, mol­ecules are linked by weak C—H⋯S hydrogen bonds and C—H⋯π inter­actions, forming layers parallel to the *bc* plane. The layers are linked *via* weak C—H⋯O hydrogen bonds and slipped parallel π–π inter­actions [inter­centroid distance = 3.6729 (10) Å], forming a three-dimensional structure. The thio­phene ring has an approximate 180° rotational disorder about the bridging C—C bond.

## Abstract 2

In the crystal of the title compound, a novel biologically active agent based on 1,3,4-oxa­diazole, mol­ecules are linked by C—H⋯S hydrogen bonds and C—H⋯π inter­actions to form layers in the *bc* plane.

## Chemical context   

1,3,4-Oxa­diazole derivatives are structural motifs of particular value in material sciences (Zhang *et al.*, 2011 ▸) and agrochemistry (Shi *et al.*, 2001 ▸; Milinkevich *et al.*, 2009 ▸; Li *et al.*, 2014 ▸). In addition, they occupy a unique situation in the field of medicinal chemistry as pharmacophores possessing diverse pharmacological activities including anti­bacterial (Ogata *et al.*, 1971 ▸; Rane *et al.*, 2012 ▸; Al-Omar, 2010 ▸), anti­cancer (Pinna *et al.*, 2009 ▸; Gamal El-Din *et al.*, 2015 ▸; Zhang *et al.*, 2014 ▸; Du *et al.*, 2013 ▸), anti­viral (Summa *et al.*, 2008 ▸; Wu *et al.*, 2015 ▸; El-Emam *et al.*, 2004 ▸), anti­hypertensive (Vardan *et al.*, 1983 ▸; Schlecker & Thieme, 1988 ▸), anti-inflammatory (Bansal *et al.*, 2014 ▸; Kadi *et al.*, 2007 ▸) and anti-oxidant (Ma *et al.*, 2013 ▸) activities. In continuation to our previous studies on 1,3,4-oxa­diazo­les (El-Emam *et al.*, 2012 ▸), we report herein on the synthesis and crystal structure of the title compound.
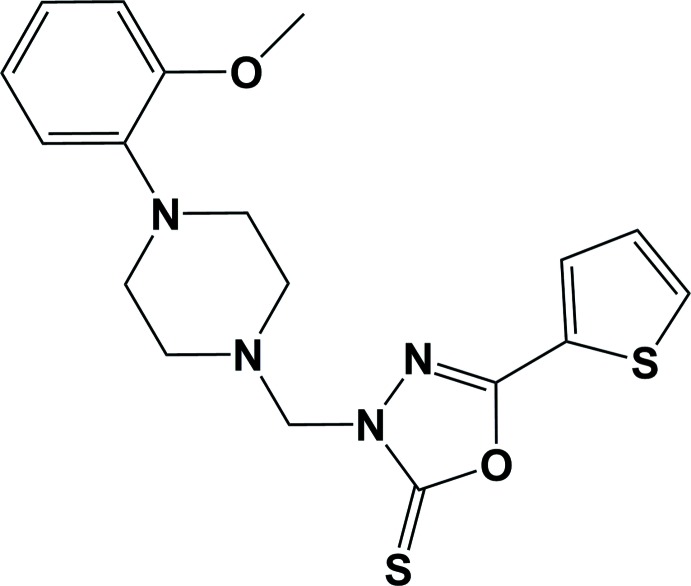


## Structural commentary   

**Figure 1:**
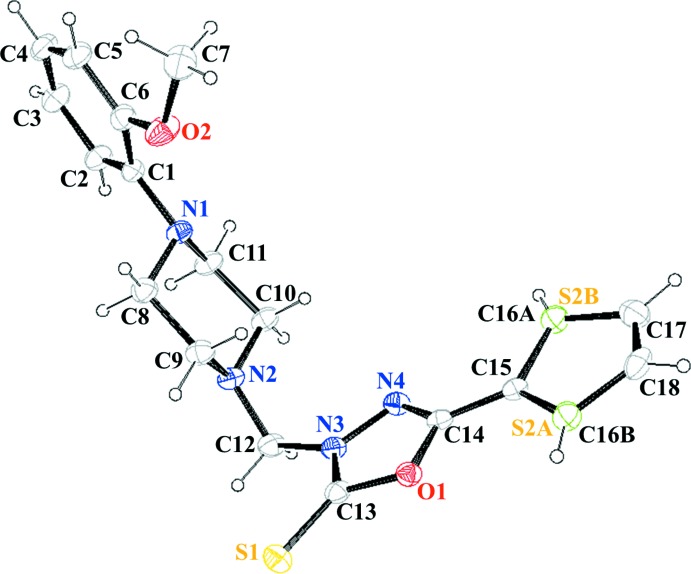
The mol­ecular structure of the title compound, showing the atom-labelling scheme and displacement ellipsoids at the 50% probability level. The thio­phene ring has an approximate 180° rotational disorder about the bridging C—C bond.

The title compound, Fig. 1 ▸, is composed of a meth­yl(thio­phen-2-yl)-1,3,4-oxa­diazole-2(3*H*)-thione moiety linked to a 2-meth­oxy­phenyl unit *via* a bridging piperazine ring. The mol­ecule is V-shaped with the mean plane of the piperazine ring, that has a chair conformation, making dihedral angles of 51.2 (1) and 77.8 (1)° with the 2-meth­oxy­phenyl ring and the oxa­diazole ring, respectively. The thio­phene ring mean plane lies almost in the plane of the oxa­diazole ring, with a dihedral angle of 4.35 (9)°. The thio­phene ring has an approximate 180° rotational disorder about the bridging C14—C15 bond.

## Supra­molecular features   

**Figure 2:**
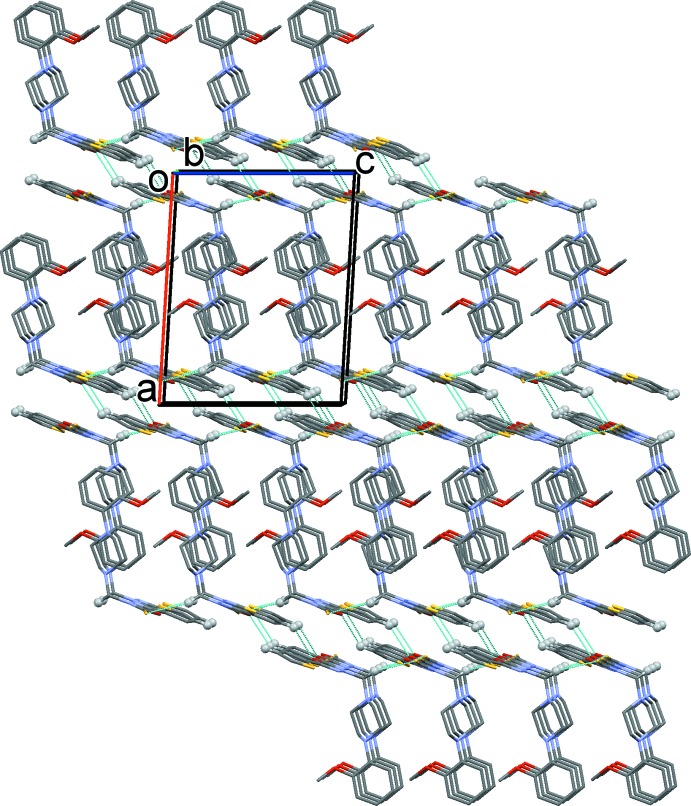
Crystal packing of the title compound, viewed along the *b* axis, showing the C—H⋯S and C—H⋯O hydrogen bonds (Table 1 ▸) as dashed lines. Only H atoms involved in inter­molecular inter­actions have been included.

In the crystal, mol­ecules are linked by weak C—H⋯S hydrogen bonds and C—H⋯π inter­actions, forming layers in the *bc* plane (Table 1 ▸ and Fig. 2 ▸). The layers are linked *via* C—H⋯O hydrogen bonds and slipped parallel π–π inter­actions [*Cg*3⋯ *Cg*1^i^ = 3.6729 (10) Å, inter-planar distance = 3.4757 (7) Å, slippage = 0.967 Å; *Cg*1 and *Cg*3 are the centroids of the S2*A*/C15/C16*A*/C17/C18 and O1/ N3/N4/C13/C14 rings, respectively; symmetry code (i): −*x* + 2, −*y* + 1, −*z* + 2], forming a three-dimensional structure (Table 1 ▸ and Fig. 2 ▸).

## Database survey   

A search of the Cambridge Structural Database (Version 5.37, last update November 2015; Groom & Allen, 2014 ▸) for the 3-methyl-5-(thio­phen-2-yl)-1,3,4-oxa­diazole-2(3*H*)-thione moiety of the title compound gave three hits. Two of these compounds also contain a substituted piperazine ring, namely 3-[(4-phenyl­piperazin-1-yl)meth­yl]-5-(2-thien­yl)-1,3,4-oxadiazole-2(3*H*)-thione (IDOBUA; El-Emam *et al.*, 2013 ▸) and 3-[(4-benzyl­piperazin-1-yl)meth­yl]-5-(thio­phen-2-yl)-2,3-dihydro-1,3,4-oxa­diazole-2-thione (VUBYUO; Al-Omary *et al.*, 2015 ▸). In both of these mol­ecules, the conformation is very similar to that of the title compound.

## Synthesis and crystallization   

To a solution of 5-(thio­phen-2-yl)-1,3,4-oxa­diazole-2-thiol (920 mg, 5 mmol), in ethanol (15 ml), 1-(2-meth­oxy­phen­yl)piperazine (960 mg, 5 mmol) and 37% formaldehyde solution (1.0 ml) were added and the mixture was stirred at room temperature for 3 h and then allowed to stand overnight at room temperature. The precipitated crude product was filtered, washed with cold ethanol, dried, and crystallized from ethanol to yield the title compound as pale-yellow prismatic crystals(yield 1.67 g, 86%; m.p. 419–421 K). Single crystals suitable for X-ray analysis were obtained by slow evaporation of a CHCl_3_:EtOH solution (1:1; 15 ml) at room temperature. ^1^H NMR (CDCl_3_, 500.13 MHz): δ 3.10 (*s*, 8H, piperazine-H), 3.85 (*s*, 3H, OCH_3_), 5.15 (*s*, 2H, CH2), 6.85–6.87 (*m*, 1H, Ar-H), 6.92–6.95 (*m*, 2H, Ar-H), 7.01–7.03 (*m*, 1H, Ar-H), 7.18 (*t*, 1H, thio­phene-H, *J* = 4.5 Hz), 7.59 (d, 1H, thio­phene-H, *J* = 4.5 Hz), 7.75 (*d*, 1H, thio­phene-H, *J* = 4.5 Hz). ^13^C NMR (CDCl_3_, 125.76 MHz): δ 50.43, 50.64 (piperazine-C), 55.33 (OCH_3_), 70.44 (CH2), 111.05, 118.28, 120.94, 123.17, 123.68, 128.32, 130.74, 130.95, 141.09, 152.23 (Ar & thio­phene-C), 155.42 (C=N), 177.74 (C=S).

## Refinement   

Crystal data, data collection and structure refinement details are summarized in Table 2 ▸. The C-bound H atoms were positioned geometrically and treated as riding atoms: C—H 0.95–0.97 Å with U_iso_(H) = 1.5*U*_eq_(C-meth­yl) and 1.2*U*_eq_(C) for other H atoms. The thienyl ring is disordered over two positions and in the final refinement cycles, the occupancy of atoms *S2*A** and C16*A*, and S2*B* and C16*B*, were each fixed at 0.5.

## Supplementary Material

Crystal structure: contains datablock(s) global, I. DOI: 10.1107/S2056989016000992/su5269sup1.cif

Structure factors: contains datablock(s) I. DOI: 10.1107/S2056989016000992/su5269Isup2.hkl

Supporting information file. DOI: 10.1107/S2056989016000992/su5269Isup3.cml

CCDC reference: 1447823

Additional supporting information:  crystallographic information; 3D view; checkCIF report

## Figures and Tables

**Table 1:** Hydrogen-bond geometry (Å, °) *Cg*1 is the centroid of the S2*A*/C15/C16*A*/C17/C18 ring.

*D*—H⋯*A*	*D*—H	H⋯*A*	*D*⋯*A*	*D*—H⋯*A*
C12—H12*A*⋯S1^i^	0.97	2.95	3.860 (2)	157
C17—H17⋯O1^ii^	0.93	2.69	3.475 (2)	143
C5—H5⋯*Cg*1^iii^	0.93	2.95	3.660 (2)	135

Symmetry codes: (i) 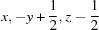; (ii) 
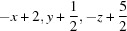
; (iii) 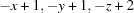.

**Table 2:** Experimental details

Crystal data	
Chemical formula	C_18_H_20_N_4_O_2_S_2_
*M*_r_	388.5
Crystal system, space group	Monoclinic, *P*2_1_/*c*
Temperature (K)	100
*a*, *b*, *c* (Å)	15.2925 (2), 10.0745 (1), 11.9726 (1)
β (°)	93.413 (1)
*V* (Å^3^)	1841.28 (3)
*Z*	4
Radiation type	Cu *K*α
μ (mm^−1^)	2.80
Crystal size (mm)	0.70 × 0.51 × 0.41

Data collection	
Diffractometer	Agilent Xcalibur Ruby Gemini
Absorption correction	Multi-scan (*CrysAlis PRO*; Agilent, 2014 ▸)
*T*_min_, *T*_max_	0.225, 0.315
No. of measured, independent and observed [*I* > 2σ(*I*)] reflections	13494, 3545, 3401
*R*_int_	0.026
(sin θ/λ)_max_ (Å^−1^)	0.612

Refinement	
*R*[*F* ^2^ > 2σ(*F* ^2^)], *wR*(*F* ^2^), *S*	0.045, 0.113, 1.04
No. of reflections	3494
No. of parameters	230
H-atom treatment	H-atom parameters constrained
Δρ_max_, Δρ_min_ (e Å^−3^)	0.95, −0.65

Computer programs: *CrysAlis CCD* and *CrysAlis RED* (Oxford Diffraction, 2006 ▸), *SHELXS97* and *SHELXL97* (Sheldrick, 2008 ▸), *ORTEP-3 for Windows* and *WinGX* (Farrugia, 2012 ▸), *Mercury* (Macrae *et al.*, 2008 ▸) and *publCIF* (Westrip, 2010 ▸).

## supplementary crystallographic information

### Crystal data

**Table:**

C_18_H_20_N_4_O_2_S_2_	*F*(000) = 816
*M**_r_* = 388.5	*D*_x_ = 1.401 Mg m^−^^3^
Monoclinic, *P*2_1_/*c*	Cu *K*α radiation, λ = 1.54184 Å
Hall symbol: -P 2ybc	Cell parameters from 11296 reflections
*a* = 15.2925 (2) Å	θ = 3.7–70.5°
*b* = 10.0745 (1) Å	µ = 2.80 mm^−^^1^
*c* = 11.9726 (1) Å	*T* = 100 K
β = 93.413 (1)°	Prism, colourless
*V* = 1841.28 (3) Å^3^	0.70 × 0.51 × 0.41 mm
*Z* = 4	

### Data collection

**Table:**

Agilent Xcalibur Ruby Gemini diffractometer	3545 independent reflections
Radiation source: Enhance (Cu) X-ray Source	3401 reflections with *I* > 2σ(*I*)
Graphite monochromator	*R*_int_ = 0.026
Detector resolution: 10.2673 pixels mm^-1^	θ_max_ = 70.7°, θ_min_ = 5.3°
ω scans	*h* = −18→17
Absorption correction: multi-scan (*CrysAlis PRO*; Agilent, 2014)	*k* = −8→12
*T*_min_ = 0.225, *T*_max_ = 0.315	*l* = −14→14
13494 measured reflections	

### Refinement

**Table:**

Refinement on *F*^2^	Primary atom site location: structure-invariant direct methods
Least-squares matrix: full	Secondary atom site location: difference Fourier map
*R*[*F*^2^ > 2σ(*F*^2^)] = 0.045	Hydrogen site location: inferred from neighbouring sites
*wR*(*F*^2^) = 0.113	H-atom parameters constrained
*S* = 1.04	*w* = 1/[σ^2^(*F*_o_^2^) + (0.0574*P*)^2^ + 2.048*P*] where *P* = (*F*_o_^2^ + 2*F*_c_^2^)/3
3494 reflections	(Δ/σ)_max_ < 0.001
230 parameters	Δρ_max_ = 0.95 e Å^−^^3^
0 restraints	Δρ_min_ = −0.65 e Å^−^^3^
0 constraints	

### Special details

**Table:**

Geometry. All e.s.d.'s (except the e.s.d. in the dihedral angle between two l.s. planes) are estimated using the full covariance matrix. The cell e.s.d.'s are taken into account individually in the estimation of e.s.d.'s in distances, angles and torsion angles; correlations between e.s.d.'s in cell parameters are only used when they are defined by crystal symmetry. An approximate (isotropic) treatment of cell e.s.d.'s is used for estimating e.s.d.'s involving l.s. planes.
Refinement. Refinement of *F*^2^ against ALL reflections. The weighted *R*-factor *wR* and goodness of fit *S* are based on *F*^2^, conventional *R*-factors *R* are based on *F*, with *F* set to zero for negative *F*^2^. The threshold expression of *F*^2^ > σ(*F*^2^) is used only for calculating *R*-factors(gt) *etc*. and is not relevant to the choice of reflections for refinement. *R*-factors based on *F*^2^ are statistically about twice as large as those based on *F*, and *R*- factors based on ALL data will be even larger.

### Fractional atomic coordinates and isotropic or equivalent isotropic displacement parameters (Å^2^)

**Table:**

	*x*	*y*	*z*	*U*_iso_*/*U*_eq_	Occ. (<1)
S1	0.89998 (3)	0.09896 (5)	0.99378 (4)	0.02414 (15)	
S2A	0.87403 (4)	0.74266 (6)	1.04820 (5)	0.02497 (16)	0.7913 (14)
C16A	0.94021 (8)	0.57554 (13)	1.21094 (10)	0.02497 (16)	0.7913 (14)
H16A	0.9598	0.501	1.2508	0.03*	0.7913 (14)
S2B	0.94021 (8)	0.57554 (13)	1.21094 (10)	0.02497 (16)	0.2087 (14)
C16B	0.87403 (4)	0.74266 (6)	1.04820 (5)	0.02497 (16)	0.2087 (14)
H16B	0.8502	0.779	0.9817	0.03*	0.2087 (14)
O1	0.90631 (8)	0.35048 (13)	1.06190 (11)	0.0189 (3)	
O2	0.43793 (9)	0.38449 (17)	0.90935 (12)	0.0312 (4)	
N3	0.85646 (10)	0.33316 (16)	0.89001 (13)	0.0192 (3)	
N1	0.55319 (10)	0.37224 (16)	0.74811 (13)	0.0198 (3)	
N4	0.85770 (10)	0.46847 (16)	0.91387 (13)	0.0204 (3)	
N2	0.73225 (10)	0.29806 (17)	0.75238 (13)	0.0210 (3)	
C1	0.46191 (12)	0.37565 (18)	0.71654 (16)	0.0202 (4)	
C15	0.90176 (11)	0.58606 (19)	1.08927 (15)	0.0183 (4)	
C14	0.88737 (11)	0.47285 (19)	1.01696 (15)	0.0180 (4)	
C13	0.88567 (12)	0.26006 (19)	0.97769 (15)	0.0187 (4)	
C6	0.40227 (13)	0.3813 (2)	0.80197 (17)	0.0229 (4)	
C9	0.67626 (13)	0.2461 (2)	0.83683 (17)	0.0237 (4)	
H9A	0.6806	0.3025	0.9026	0.028*	
H9B	0.6955	0.1577	0.8589	0.028*	
C11	0.61058 (12)	0.4202 (2)	0.66366 (16)	0.0231 (4)	
H11A	0.5908	0.5066	0.6368	0.028*	
H11B	0.6088	0.3596	0.6007	0.028*	
C4	0.28120 (13)	0.3853 (2)	0.66375 (19)	0.0278 (5)	
H4	0.2211	0.3875	0.6463	0.033*	
C3	0.33874 (14)	0.3826 (2)	0.57913 (18)	0.0284 (5)	
H3	0.3177	0.3844	0.5046	0.034*	
C2	0.42871 (13)	0.3770 (2)	0.60627 (17)	0.0249 (4)	
H2	0.4672	0.3741	0.5491	0.03*	
C10	0.70379 (12)	0.4304 (2)	0.71497 (16)	0.0228 (4)	
H10A	0.7424	0.4638	0.66	0.027*	
H10B	0.7058	0.4912	0.7779	0.027*	
C8	0.58162 (13)	0.2411 (2)	0.78978 (18)	0.0240 (4)	
H8A	0.5763	0.177	0.7292	0.029*	
H8B	0.5441	0.2125	0.8478	0.029*	
C5	0.31283 (13)	0.3849 (2)	0.77469 (18)	0.0275 (5)	
H5	0.2738	0.3869	0.8313	0.033*	
C12	0.82390 (12)	0.2821 (2)	0.77866 (15)	0.0224 (4)	
H12A	0.8555	0.3268	0.7217	0.027*	
H12B	0.8379	0.1883	0.7749	0.027*	
C17	0.93757 (13)	0.7167 (2)	1.24746 (17)	0.0268 (4)	
H17	0.9571	0.7421	1.3193	0.032*	
C7	0.38537 (15)	0.4430 (3)	0.99152 (19)	0.0342 (5)	
H7A	0.4167	0.4403	1.0634	0.051*	
H7B	0.3316	0.3943	0.9947	0.051*	
H7C	0.3726	0.5335	0.9715	0.051*	
C18	0.90558 (13)	0.8060 (2)	1.1716 (2)	0.0297 (5)	
H18	0.9016	0.8961	1.1874	0.036*	

### Atomic displacement parameters (Å^2^)

**Table:**

	*U*^11^	*U*^22^	*U*^33^	*U*^12^	*U*^13^	*U*^23^
S1	0.0282 (3)	0.0197 (3)	0.0246 (3)	0.00178 (18)	0.00181 (19)	0.00051 (18)
S2A	0.0229 (3)	0.0257 (3)	0.0265 (3)	−0.0011 (2)	0.0022 (2)	−0.0008 (2)
C16A	0.0229 (3)	0.0257 (3)	0.0265 (3)	−0.0011 (2)	0.0022 (2)	−0.0008 (2)
S2B	0.0229 (3)	0.0257 (3)	0.0265 (3)	−0.0011 (2)	0.0022 (2)	−0.0008 (2)
C16B	0.0229 (3)	0.0257 (3)	0.0265 (3)	−0.0011 (2)	0.0022 (2)	−0.0008 (2)
O1	0.0189 (6)	0.0199 (6)	0.0177 (6)	0.0015 (5)	0.0001 (5)	0.0003 (5)
O2	0.0213 (7)	0.0510 (10)	0.0218 (7)	−0.0010 (6)	0.0057 (6)	−0.0044 (6)
N3	0.0175 (7)	0.0216 (8)	0.0184 (8)	0.0028 (6)	0.0006 (6)	−0.0007 (6)
N1	0.0161 (8)	0.0234 (8)	0.0201 (8)	0.0005 (6)	0.0037 (6)	0.0027 (6)
N4	0.0196 (8)	0.0219 (8)	0.0197 (8)	0.0030 (6)	0.0022 (6)	0.0002 (6)
N2	0.0170 (8)	0.0260 (8)	0.0201 (8)	0.0035 (6)	0.0011 (6)	0.0002 (7)
C1	0.0175 (9)	0.0190 (9)	0.0244 (10)	−0.0012 (7)	0.0030 (7)	0.0003 (7)
C15	0.0141 (8)	0.0219 (9)	0.0191 (9)	0.0009 (7)	0.0019 (7)	0.0014 (7)
C14	0.0128 (8)	0.0209 (9)	0.0204 (9)	0.0022 (7)	0.0029 (7)	0.0023 (7)
C13	0.0143 (8)	0.0241 (10)	0.0182 (9)	0.0006 (7)	0.0031 (7)	−0.0016 (7)
C6	0.0216 (10)	0.0233 (10)	0.0239 (10)	−0.0022 (8)	0.0034 (8)	0.0000 (8)
C9	0.0218 (10)	0.0248 (10)	0.0245 (10)	0.0031 (8)	0.0021 (8)	0.0047 (8)
C11	0.0177 (9)	0.0309 (11)	0.0211 (9)	0.0008 (8)	0.0031 (7)	0.0060 (8)
C4	0.0163 (9)	0.0309 (11)	0.0360 (11)	−0.0025 (8)	0.0000 (8)	0.0014 (9)
C3	0.0228 (10)	0.0351 (12)	0.0267 (10)	−0.0024 (8)	−0.0019 (8)	0.0032 (9)
C2	0.0197 (9)	0.0309 (11)	0.0246 (10)	−0.0008 (8)	0.0042 (8)	0.0010 (8)
C10	0.0177 (9)	0.0284 (10)	0.0224 (9)	−0.0002 (8)	0.0024 (7)	0.0055 (8)
C8	0.0203 (9)	0.0228 (10)	0.0290 (10)	−0.0003 (7)	0.0030 (8)	0.0035 (8)
C5	0.0197 (10)	0.0331 (11)	0.0307 (11)	−0.0032 (8)	0.0087 (8)	−0.0001 (9)
C12	0.0204 (9)	0.0295 (10)	0.0174 (9)	0.0047 (8)	0.0017 (7)	−0.0042 (8)
C17	0.0173 (9)	0.0420 (12)	0.0211 (9)	−0.0069 (8)	0.0027 (7)	−0.0057 (9)
C7	0.0326 (11)	0.0442 (13)	0.0272 (11)	−0.0067 (10)	0.0117 (9)	−0.0083 (10)
C18	0.0240 (10)	0.0230 (10)	0.0432 (12)	−0.0017 (8)	0.0102 (9)	−0.0017 (9)

### Geometric parameters (Å, º)

**Table:**

S1—C13	1.647 (2)	C9—H9A	0.97
S2A—C18	1.655 (2)	C9—H9B	0.97
S2A—C15	1.6988 (19)	C11—C10	1.522 (3)
C16A—C17	1.489 (3)	C11—H11A	0.97
C16A—C15	1.542 (2)	C11—H11B	0.97
C16A—H16A	0.93	C4—C3	1.381 (3)
O1—C14	1.369 (2)	C4—C5	1.386 (3)
O1—C13	1.381 (2)	C4—H4	0.93
O2—C6	1.367 (3)	C3—C2	1.396 (3)
O2—C7	1.434 (3)	C3—H3	0.93
N3—C13	1.337 (2)	C2—H2	0.93
N3—N4	1.393 (2)	C10—H10A	0.97
N3—C12	1.487 (2)	C10—H10B	0.97
N1—C1	1.425 (2)	C8—H8A	0.97
N1—C11	1.460 (2)	C8—H8B	0.97
N1—C8	1.469 (2)	C5—H5	0.93
N4—C14	1.290 (2)	C12—H12A	0.97
N2—C12	1.427 (2)	C12—H12B	0.97
N2—C9	1.460 (2)	C17—C18	1.349 (3)
N2—C10	1.464 (3)	C17—H17	0.93
C1—C2	1.386 (3)	C7—H7A	0.96
C1—C6	1.412 (3)	C7—H7B	0.96
C15—C14	1.441 (3)	C7—H7C	0.96
C6—C5	1.388 (3)	C18—H18	0.93
C9—C8	1.522 (3)		
			
C18—S2A—C15	92.58 (10)	C3—C4—C5	120.10 (19)
C17—C16A—C15	101.32 (13)	C3—C4—H4	120
C17—C16A—H16A	129.3	C5—C4—H4	120
C15—C16A—H16A	129.3	C4—C3—C2	119.49 (19)
C14—O1—C13	105.85 (14)	C4—C3—H3	120.3
C6—O2—C7	116.52 (17)	C2—C3—H3	120.3
C13—N3—N4	112.23 (15)	C1—C2—C3	121.48 (19)
C13—N3—C12	126.27 (17)	C1—C2—H2	119.3
N4—N3—C12	121.49 (15)	C3—C2—H2	119.3
C1—N1—C11	115.34 (15)	N2—C10—C11	108.46 (16)
C1—N1—C8	112.16 (15)	N2—C10—H10A	110
C11—N1—C8	110.80 (15)	C11—C10—H10A	110
C14—N4—N3	103.26 (15)	N2—C10—H10B	110
C12—N2—C9	114.55 (15)	C11—C10—H10B	110
C12—N2—C10	116.12 (16)	H10A—C10—H10B	108.4
C9—N2—C10	111.25 (15)	N1—C8—C9	110.57 (16)
C2—C1—C6	118.29 (18)	N1—C8—H8A	109.5
C2—C1—N1	123.40 (17)	C9—C8—H8A	109.5
C6—C1—N1	118.28 (17)	N1—C8—H8B	109.5
C14—C15—C16A	123.32 (15)	C9—C8—H8B	109.5
C14—C15—S2A	122.32 (14)	H8A—C8—H8B	108.1
C16A—C15—S2A	114.33 (12)	C4—C5—C6	120.56 (19)
N4—C14—O1	113.53 (16)	C4—C5—H5	119.7
N4—C14—C15	129.37 (18)	C6—C5—H5	119.7
O1—C14—C15	117.09 (16)	N2—C12—N3	115.51 (15)
N3—C13—O1	105.12 (16)	N2—C12—H12A	108.4
N3—C13—S1	132.09 (15)	N3—C12—H12A	108.4
O1—C13—S1	122.77 (14)	N2—C12—H12B	108.4
O2—C6—C5	123.60 (18)	N3—C12—H12B	108.4
O2—C6—C1	116.35 (17)	H12A—C12—H12B	107.5
C5—C6—C1	120.05 (19)	C18—C17—C16A	117.01 (18)
N2—C9—C8	109.84 (16)	C18—C17—H17	121.5
N2—C9—H9A	109.7	C16A—C17—H17	121.5
C8—C9—H9A	109.7	O2—C7—H7A	109.5
N2—C9—H9B	109.7	O2—C7—H7B	109.5
C8—C9—H9B	109.7	H7A—C7—H7B	109.5
H9A—C9—H9B	108.2	O2—C7—H7C	109.5
N1—C11—C10	109.26 (15)	H7A—C7—H7C	109.5
N1—C11—H11A	109.8	H7B—C7—H7C	109.5
C10—C11—H11A	109.8	C17—C18—S2A	114.76 (17)
N1—C11—H11B	109.8	C17—C18—H18	122.6
C10—C11—H11B	109.8	S2A—C18—H18	122.6
H11A—C11—H11B	108.3		
			
C13—N3—N4—C14	−0.5 (2)	N1—C1—C6—O2	0.7 (3)
C12—N3—N4—C14	178.38 (15)	C2—C1—C6—C5	1.5 (3)
C11—N1—C1—C2	22.5 (3)	N1—C1—C6—C5	179.97 (18)
C8—N1—C1—C2	−105.7 (2)	C12—N2—C9—C8	−167.82 (16)
C11—N1—C1—C6	−155.90 (18)	C10—N2—C9—C8	58.0 (2)
C8—N1—C1—C6	76.0 (2)	C1—N1—C11—C10	171.49 (16)
C17—C16A—C15—C14	−177.76 (16)	C8—N1—C11—C10	−59.7 (2)
C17—C16A—C15—S2A	0.16 (15)	C5—C4—C3—C2	1.1 (3)
C18—S2A—C15—C14	177.79 (16)	C6—C1—C2—C3	−0.5 (3)
C18—S2A—C15—C16A	−0.16 (13)	N1—C1—C2—C3	−178.91 (19)
N3—N4—C14—O1	0.53 (19)	C4—C3—C2—C1	−0.8 (3)
N3—N4—C14—C15	−178.64 (17)	C12—N2—C10—C11	165.92 (15)
C13—O1—C14—N4	−0.4 (2)	C9—N2—C10—C11	−60.7 (2)
C13—O1—C14—C15	178.90 (15)	N1—C11—C10—N2	60.7 (2)
C16A—C15—C14—N4	−178.37 (16)	C1—N1—C8—C9	−172.53 (16)
S2A—C15—C14—N4	3.9 (3)	C11—N1—C8—C9	57.0 (2)
C16A—C15—C14—O1	2.5 (2)	N2—C9—C8—N1	−55.2 (2)
S2A—C15—C14—O1	−175.28 (13)	C3—C4—C5—C6	−0.2 (3)
N4—N3—C13—O1	0.29 (19)	O2—C6—C5—C4	178.1 (2)
C12—N3—C13—O1	−178.53 (15)	C1—C6—C5—C4	−1.2 (3)
N4—N3—C13—S1	−178.25 (15)	C9—N2—C12—N3	−52.8 (2)
C12—N3—C13—S1	2.9 (3)	C10—N2—C12—N3	79.1 (2)
C14—O1—C13—N3	0.03 (18)	C13—N3—C12—N2	110.8 (2)
C14—O1—C13—S1	178.74 (13)	N4—N3—C12—N2	−67.9 (2)
C7—O2—C6—C5	−24.0 (3)	C15—C16A—C17—C18	−0.1 (2)
C7—O2—C6—C1	155.31 (19)	C16A—C17—C18—S2A	0.0 (2)
C2—C1—C6—O2	−177.78 (18)	C15—S2A—C18—C17	0.11 (17)

### Hydrogen-bond geometry (Å, º)

Cg1 is the centroid of the S2*A*/C15/C16*A*/C17/C18 ring.

**Table:**

*D*—H···*A*	*D*—H	H···*A*	*D*···*A*	*D*—H···*A*
C12—H12*A*···S1^i^	0.97	2.95	3.860 (2)	157
C17—H17···O1^ii^	0.93	2.69	3.475 (2)	143
C5—H5···*Cg*1^iii^	0.93	2.95	3.660 (2)	135

Symmetry codes: (i) *x*, −*y*+1/2, *z*−1/2; (ii) −*x*+2, *y*+1/2, −*z*+5/2; (iii) −*x*+1, −*y*+1, −*z*+2.
